# A slight degree of osteoarthritis appears to be present after anterior cruciate ligament reconstruction compared with contralateral healthy knees at a minimum of 20 years: A systematic review of the literature

**DOI:** 10.1002/jeo2.12017

**Published:** 2024-04-04

**Authors:** Riccardo D'Ambrosi, Alessandro Carrozzo, Amit Meena, Katia Corona, Amit Kumar Yadav, Alessandro Annibaldi, Srinivas B. S. Kambhampati, Elisabeth Abermann, Christian Fink

**Affiliations:** ^1^ IRCCS Ospedale Galeazzi—Sant'Ambrogio Milan Italy; ^2^ Dipartimento di Scienze Biomediche per la Salute Università degli Studi di Milano Milan Italy; ^3^ Orthopaedic Unit, Sant'Andrea Hospital University of Rome La Sapienza Rome Italy; ^4^ Division of Orthopedics Shalby Multi‐Specialty Hospital Jaipur India; ^5^ Gelenkpunkt—Sports and Joint Surgery FIFA Medical Centre of Excellence Innsbruck Austria; ^6^ Department of Medicine and Health Sciences “Vincenzo Tiberio” University of Molise Campobasso Italy; ^7^ Wrightington Hospital Wigan UK; ^8^ Sri Dhaatri Orthopaedic Maternity & Gynaecology Center, SKDGOC Vijayawada Andhra Pradesh India; ^9^ Research Unit for Orthopaedic Sports Medicine and Injury Prevention (OSMI), Private University for Health Sciences Medical Informatics and Technology Innsbruck Austria

**Keywords:** Ahlbäck, anterior cruciate ligament, failure rate, IKDC, Kellgren–Lawrence, kneeosteoarthritisradiographic

## Abstract

**Purpose:**

The aim of the present systematic review was to quantitatively synthesize the best literature evidence regarding osteoarthritis developing after anterior cruciate ligament reconstruction (ACLR), including only studies with a follow‐up duration of at least 20 years.

**Material and Methods:**

A systematic review was conducted based on the Preferred Reporting Items for Systematic Reviews and Meta‐Analyses (PRISMA) guidelines on four electronic databases (PubMed, Scopus, EMBASE and Cochrane Library). The outcome measures extracted from the studies were failure rate, subsequent knee surgery on the same knee, radiographic development of osteoarthritis measured with Kellgren–Lawrence, International Knee Documentation Committee (IKDC) radiographic score and Ahlbäck classification. The health of both the ACLR knee and the contralateral knee was compared.

**Results:**

A total of 1552 patients were included in the study, of which 1290 (83.11%) were operated on using a patellar tendon graft, 190 (12.24%) with hamstrings, 27 (1.73%) with an iliotibial band and 45 (2.89%) with patellar tendon plus a ligament augmentation device (LAD). The mean age at the time of surgery was 25.18 ± 1.91 years, and the mean follow‐up time was 23.34 ± 2.56 years. Analysing IDKC Score at final follow‐up, ACLR Group showed a higher degree of OA compared with contralateral healthy knee (*p* < 0.01), but only 33.2% (324/976) of the patients showed a moderate to severe degree (Grade C or D) of osteoarthritis, while for Kellgren–Lawrence, ACLR Group showed a higher degree of OA compared with contralateral healthy knee (*p* < 0.01), but only 28.9% (196/678) of the patients showed a moderate to severe degree (Grade III or IV) of osteoarthritis. In total, 1552 patients were registered, 155 reruptures (9.98%) and a total of 300 (19.3%) new surgeries, of which 228 meniscectomy (14.69%), 21 (1.35%) knee arthroplasty and 17 (1.09%) hardware removal were recorded.

**Conclusions:**

ACL reconstruction appears to result in mild osteoarthritis in the long term in most of the patients and only less than 33.2% develop a moderate to severe degree of knee OA according to IKDC radiographic score. A slight degree of osteoarthritis appears to be present in ACLR knees compared with contralateral healthy knees.

**Level of Evidence:**

Level IV.

**Abbreviations**
ACL: anterior cruciate ligament
ACLR: anterior cruciate ligament reconstruction
HT: hamstrings
IKDC: International Knee Documentation Committee radiographic score
ITB: iliotibial band
LAD: Ligament Augmentation Device
LET: lateral extrarticular tenodesis
OA: osteoarthritis
PT: patellar tendon

## INTRODUCTION

ACL tears are frequently observed in young and physically active individuals who engage in sports activities characterized by contact, deceleration, twisting, cutting and jumping [25]. It has been reported that approximately 200,000 ACL tears occur in the United States annually [23]. Moreover, there has been a notable increase in the frequency of ACL reconstructions, with rates increasing from 32.4 patients per 100,000 person/year in the early 1990s to 43.5 patients per 100,000 person/year in the 2010s [3]. The presence of laxity resulting from an ACL injury leads to a decrease in knee functionality and disrupts the usual balance within the joint [12]. This disruption manifests in several ways, including decreased activity levels and a diminished quality of life [9]. Although adverse biomechanical alterations have been well acknowledged, there is on‐going debate regarding their management. Various surgical and conservative therapies have been suggested thus far [1, 11, 14, 17, 18, 19, 33, 39].

However, an even more contentious issue is the potential to mitigate joint degradation by ACL reconstruction, as there are conflicting findings regarding the optimal strategy for preventing knee OA [4, 26, 31, 32, 42, 44, 45, 46].

The literature extensively documents the short‐term results of this surgery, revealing that the clinical outcomes are quite favourable for the majority of patients. These outcomes include the restoration of stability, a high rate of return to sports, and a minimal occurrence of failures. Nevertheless, the majority of research have only conducted short‐term or midterm follow‐ups, therefore failing to offer any understanding of the prolonged impact of ACLR. ACL injury is linked to changes in joint stability, and these changes in joint movement can eventually result in the development of knee OA over a prolonged period of time. Therefore, it is necessary to conduct follow‐up studies lasting more than 10 years in order to examine the factors that contribute to OA and accurately determine the occurrence of this long‐term condition [5, 20].

The aim of the present systematic review was to quantitatively synthesize the best literature evidence on this topic, including only studies in which researchers performed radiographic osteoarthritis evaluation at the 20‐year follow‐up visit after ACL reconstruction. The hypothesis was that patients who underwent surgical treatment would have a mild grade of osteoarthritis at the long‐term follow‐up compared with contralateral healthy knee, and only a small percentage of operated patients would need replacement surgery.

## MATERIAL AND METHODS

The current systematic review follows the Preferred Reporting Items for Systematic Reviews and Meta‐Analyses (PRISMA) guidelines and is registered in the PROSPERO Registry CRD42023453941 [27, 28]. The A MeaSurement Tool to Assess systematic Reviews (AMSTAR)‐2 checklist was used to confirm the quality of the systematic review [36].

### Eligibility criteria

The literature selected for this study was based on the following criteria.

#### Study design

Randomized controlled trials, controlled (nonrandomized) clinical trials, prospective and retrospective comparative cohort studies, case‒control studies and case series were included. Case reports and case series that did not report data on the radiographic development of osteoarthritis were excluded.

#### Participants

The studies were conducted on skeletally mature patients who underwent ACL reconstructions and were followed for a minimum of 20 years.

#### Interventions

Studies have reported data on the radiographic development of osteoarthritis in skeletally mature patients who underwent anterior cruciate ligament reconstruction. ACL repair or revision surgery were considered an exclusion criterion.

#### Types of outcome measures

The outcome measures extracted from the studies were the failure and subsequent knee surgery on the same knee rates, radiographic development of osteoarthritis measured with Kellgren–Lawrence, IKDC radiographic score and Ahlbäck classification. If appropriate, the health of both the ACLR knee and the contralateral knee was compared. No subanalysis were performed according to concomitant procedures.

#### Follow‐up

According to the inclusion criteria, the minimum follow‐up duration was 20 years. Studies reporting longer average follow‐up times but in which the minimum follow‐up was shorter or not specified were excluded unless it was possible to identify and isolate data from the subgroup with more than 20 years of follow‐up data. Therefore, all patients considered underwent at least 20 years of follow‐up.

### Information sources and search

A systematic search for relevant literature was performed in the PubMed (MEDLINE), Scopus, EMBASE, and Cochrane Library databases of all studies published in English from January 1990 to August 2023. The search was carried out in August 2023. Two independent reviewers (R. D. and A. C.) assisted in conducting and validating the search. The following search terms were entered into the title, abstract, and keyword fields: ‘anterior cruciate ligament’ or ‘ACL’ AND ‘long‐term’ or ‘20‐year’ AND ‘reconstruction’ AND ‘osteoarthritis’ or ‘radiographic’. Finally, only papers published in English were included.

### Data collection and analysis

#### Study selection

The retrieved articles were screened by title and, if found relevant, screened further by reading the abstract. After excluding studies that did not meet the eligibility criteria, the entire content of the remaining articles was evaluated for eligibility. To minimize the risk of bias, the authors reviewed and discussed all the selected articles, references and articles excluded from the study. In case of any disagreement between the reviewers, the senior investigator made the final decision. At the end of the process, further studies that might have been missed were manually searched by going through the reference lists of the included studies and relevant systematic reviews.

#### Data collection process

The first two authors extracted the data from the selected articles using a computerized tool created with Microsoft Access (Version 2010, Microsoft Corp). Each article was validated again by the first author before analysis. For each study, patient data were extracted (age, sex), and surgical details, including surgical technique, graft type, rate of complications, revision surgeries and development of osteoarthritis, were recorded.

#### Level of evidence

The Oxford Levels of Evidence set by the Oxford Centre for Evidence‐Based Medicine were used to categorize the level of evidence [13].

#### Evaluation of the quality of studies

The quality of the selected studies was evaluated using the Methodological Index for Nonrandomized Studies (MINORS) score. The checklist includes 12 items, of which the last four are specific to comparative studies. Each item was given a score of 0–2 points. The ideal score was 16 points for noncomparative studies and 24 for comparative studies [30].

Furthermore, according to AMSTAR‐2 guidelines, every article was assessed using the ROBINS‐I tool [36].

#### Statistical analysis

The extracted quantitative parameters (age, follow‐up time and results of the radiographic scores) were given as the mean ± standard deviation (SD) or percentage when provided in the articles. Otherwise, alternative values such as the median or range were extracted. To test score differences between the groups, student's *t* test was used to evaluate differences amongst groups at the 20‐year follow‐up. All tests were two‐sided, and *p* < 0.05 was considered statistically significant. Statistical analyses were conducted in the R version. Statistical analysis was performed with Review Manager (Version 5.3, The Cochrane Collaboration). Mean difference (MD) was used as summary statistics to perform statistical analysis of continuous variables. They were reported with 95% confidence intervals (95% CI), and *p* value of 0.05 was used as the level of statistical significance.

## RESULTS

### Search results

**Figure 1:**
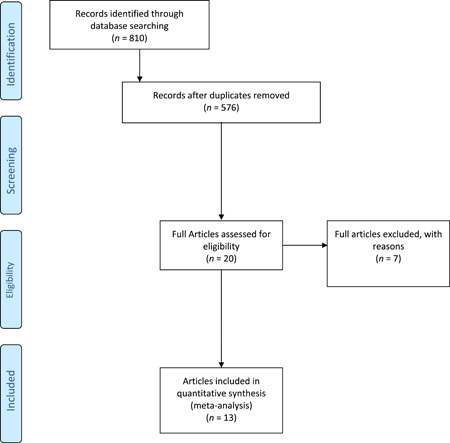
A flowchart of the literature screening performed in this study.

The electronic search yielded 810 studies. After 234 duplicates were removed, 20 studies remained, out of which seven were excluded after reviewing the abstracts, bringing the number down to 13 [8, 10, 15, 21, 24, 29, 37, 38, 40, 41, 43, 47, 48]. No additional studies were found by manually searching the reference lists of the selected articles. Figure 1 shows the flowchart depicting the selection process for the studies.

The analysed studies had a mean MINORS score of 14.76 ± 1.5, confirming the methodological quality of the available literature.

#### Patient characteristics and surgical protocol

A total of 1552 patients were included in the study, of which 1290 (83.11%) were operated on using a patellar tendon (PT) graft, 190 (12.24%) with hamstrings, 27 (1.73%) with an iliotibial band and 45 (2.89%) with PT plus a ligament augmentation device (LAD). The mean age at the time of surgery was 25.18 ± 1.91 years, and the mean follow‐up time was 23.34 ± 2.56 years. In total, 1552 patients were registered, 155 reruptures (9.98%) and a total of 300 (19.3%) new surgeries, of which 228 meniscectomy (14.69%), 21 (1.35%) knee arthroplasty and 17 (1.09%) hardware removal were recorded. Detailed results are reported in Table 1.

**Table 1:** Characteristics of the selected studies.

Authors, Year	MINORS	Level of evidence	Patients (*n*)	M:F (*n*)	Age *Mean ± SD (range)*	Graft	Surgical technique	Follow‐up	Associated injuries %, (*n*)	Failure rate %, (*n*)	Complications/subsequent surgery %, (*n*)	Osteoarthritis	Contralateral knee
Curado et al., 2019 [10]^a^	14	IV	182	67:115	26 ± 7	Patellar tendon 93% (170) Not specified 7% (12)	Arthroscopic 82% (149) Open 18% (33) ALL 7.2% (13)	22 ± 1	5% (9) Meniscal suture 31% (56) Meniscectomy	13% (24)	1.6% (3) Infections 2.2% (4) Regional pain syndrome 2.2% (4) Stiffness	IKDC grade (182): A 50% (91) B 21% (37) C 19% (36) D 10% (18)	
Costa‐Paz, et al., 2019 [8]	13	IV	76	59:13	30 (22–36)	Patellar tendon 100% (76)	100% (76) Arthroscopic	22 [21–25]	32 (45%) Meniscectomy 3 (4%) Meniscal suture	5% (4)		IKDC grade (60): A 15% (9) B 57% (34) C 18% (11) D 10% (6)	IKDC grade (41): A 51% (21) B 42% (17) C 7% (3)
Hagemans et al., 2020 [21]	15	IV	48	31:17	31 (26–39)	Hamstrings 100% (48)	Transtibial, single bundle 100% (48)	21 (20–22)	23 (48%) Meniscectomy	8% (4)	13% (6) Subsequent mensicectomy 13% (6) Staples removal 6% (3) Arthroscopy	Kelllgren–Lawrence (39): Grade 0 23% (9) Grade 1 28% (11) Grade 2 8% (3) Grade 3 28% (11) Grade 4 13% (5)	Kelllgren–Lawrence (39): Grade 0 49% (19) Grade 1 41% (16) Grade 2 10% (4)
Söderman et al., 2020 [38]	15	III	60	55:5	26 (17–47)	Patellar tendon 100% (60)	Transtibial (100%) (60)	31 (28–33)		50% (30)		Kellgren–Lawrence (60): Grade 0 Patellofemoral 23% (14) Medial 15% (9) Lateral 28% (17) Grade 1 Patellofemoral 37% (22) Medial 11% (18) Lateral 14% (24) Grade 2 Patellofemoral 9% (15) Medial 7% (11) Lateral 6% (10) Grade 3 Patellofemoral 13% (8) Medial 9% (15) Lateral 2% (4) Grade 4 Patellofemoral 1% (1) Medial 4% (7) Lateral 3% (5)	
Lindanger et al., 2022 [24]^a^	16	III	235	115:120	25.4 ± 7.1	Patellar tendon 100% (235)	Transtibial 100% (235), Mini‐open 77% (182), Arthroscopic 23% (53) Kennedy LAD 11% (25)	25 years (20–31)	16% (38) Medial meniscectomy 14% (32) Lateral meniscectomy	9% (20)	5% (11) Knee replacement surgery 15% (25) Medial meniscectomy 5% (11) Lateral Meniscectomy	Kelllgren–Lawrence (224 patients): Grade 0 20% (47) Grade 1 20% (47) Grade 2 24% (57) Grade 3 24% (57) Grade 4 7% (16)	Kelllgren–Lawrence (224): Grade 0 57% (135) Grade 1 22% (52) Grade 2 8% (18) Grade 3 7% (17) Grade 4 1% (2)
Pernin et al., 2010 [29]	13	IV	100	NA	25.1 (14.2–43.3)	Patellar tendon + ALL 100% (100)	Open 100% (100)	24.5 (21–28)	8% (8) Medial meniscal suture 27% (27) Medial meniscectomy	18% (18)	7% (7) Medial meniscectomy 8% (8) Tibial osteotomies	IKDC grade (100): A 39% (39) B 7% (7) C 27% (27) D 27% (27)	IKDC grade (100): A–B 85% (85) C–D 15% (15)
Shelbourne et al., 2017 [37]	14	II	423	287:136	23.2 ± 6.9	Patellar tendon 100% (423)		22.5 (20–33.1)	26.9% (114) Medial meniscectomy 13% (55) Lateral meniscectomy 12.3% (52) Medial and lateral meniscectomy		29.1% (123) Medial meniscectomy 4.2% (18) Lateral meniscectomy	IKDC grade (423): A Patellofemoral 63.4% (268) Medial 55.8% (236) Lateral 66.4% (281) B Patellofemoral 26% (110) Medial 25.1% (106) Lateral 22.7% (96) C Patellofemoral 9.5% (40) Medial 13% (55) lateral 7.6% (32) D Patellofemoral 1.2% (5) Medial 6.1% (26) Lateral 3.3% (14)	
Thompson et al., 2016 [41]	16	IV	180	90:90	Patellar tendon: 25 (15–42) Hamstrings 24 (13–52)	Patellar tendon 50% (90) Hamstrings 50% (90)	Arthroscopy 100% (90)	20 years	Patellar tendon: 20% (18) Medial meniscus injuries 38% (34) Lateral meniscus injuries 8% (7) Meniscal suture 7% (6) Meniscectomy Hamstrings: 22% (20) Medial meniscus injuries 48% (43) Lateral meniscus injuries 11% (10) Meniscal suture 10% (9) Meniscectomy	Patellar tendon: 10% (9) Hamstrings 18% (16)	Patellar tendon 10% (9) Meniscectomy 1% (1) Tibial screw removal 1% (1) Patellar tendon cyst 2% (2) Asportation cyclops lesion 2% (2) Arthroscopy Hamstrings: 13% (12) Meniscectomy 2% (2) Tibial screw removal 1% (1) Asportation cyclops lesion	IKDC (122 patients): A–B 83.6% (102) C–D 16.4% (20) Patellar tendon (61) A 39% (24) B 41% (25) C 20% (12) Hamstrings (61) A 59% (36) B 28% (17) C 6.5% (4) D 6.5% (4)	
Van Yperen et al., 2018 [43]	14	II	25	19:6		Patellar tendon 100% (25)	Arthroscopy 100% (25)	20	15% (6) Meniscectomy	16% (4)	4% (1) Total knee arthroplasty 16% (4) Meniscectomy	Kelllgren–Lawrence (25): Grade 0 4% (1) Grade 1 16% (4) Grade 2 64% (16) Grade 3 12% (3) Grade 4 4% (1)	
Yamaguchi et al., 2006 [47]	13	IV	27	18:9	24.8 (16–42)	Iliotibial band 100% 27	Open 27 (100%)	24	56% (15) Medial meniscectomy 3.7% (1) Lateral meniscectomy 7.4% (2) Combined medial and lateral meniscectomy 7.4% (2) MCL repair	1 (3.7%)	18.5% (5) Medial meniscectomy 15% (4) Lateral meniscecomy 3.7% (1) Medial and lateral meniscecomy 3.7% (1) Arthroscopy due to stiffness	IKDC grade (24): A 8% (2) B 21% (5) C 25% (6) D 46% (11)	IKDC grade (24 patients): A 42% (10) B 42% (10) C 8% (2) D 8% (2)
Zaffagnini et al., 2017 [48]	14	IV	52	11:41	25.5 ± 7.6	Hamstrings 100% (52)	Arthroscopy with over‐the‐top technique 52 (100%)	24	15% (8) Medial meniscectomy 15% (8) Lateral meniscectomy	10% (5) Re‐ruptures/clinical failures	8% (4) Paraesthesia 15% (8) Staples removal 6% (3) Partial medial meniscectomy 2% (1) Osteotomy	Kelllgren–Lawrence (51): Grade 0 3.5% (1) Grade 1 31% (9) Grade 2 48.2% (14) Grade 3 17.3% (5)	
Elveos et al., 2018 [15]	18	I	93		Patellar tendon: 25 (16–42) Patellar tendon + LAD: 27 (17–48)	Patellar tendon 52% (48) Patellar tendon + LAD 48% (45)	Arthroscopy 100% (93)	25 (24–26)		20% (19) Patellar tendon 23% (12) Patellar tendon + LAD 15.6% (7)	Knee arthroplasty 8% (7) 2.1% (1) in patellar tendon 13.3% (6) in patellar tendon + LAD	Ahlbäck Patellar tendon (28) Grade 0 0% (0) Grade I 68% (19) Grade II 0% (0) Grade III 29% (8) Grade IV 3% (1) Patellar tendon + LAD (28) Grade 0 0% (0) Grade I 68% (19) Grade II 11% (3) Grade III 18% (5) Grade IV 3% (1) Grade V 0% (0)	
Sporshelm et al., 2019 [40]	17	I	51			Patellar tendon 100% (51)	Open 51 (100%)	30 (29–31)		2% (1)	4% (2) Knee arthroplasty	Ahlbäck (26) Grade 0 0% (0) Grade I 55%% (12) Grade II 38% (10) Grade III 15% (4) Grade IV 0% (0) Grade V 0% (0)	Ahlbäck (26) Grade 0 4% (1) Grade I 77% (20) Grade II 15% (4) Grade III 4% (1) Grade IV 0% (0) Grade V 0% (0)

Abbreviations: IKDC, International Knee Documentation Committee; LAD, ligament augmentation device; MCL, medial collateral ligament; NA, not available.

^a^ Where graft and results by subgroup are not specified, is considered to be the one primarily used in the study.

### Radiographic results

#### IKDC Radiographic Score

Analysing IDKC Score at final follow‐up, ACLR Group showed a higher degree of OA compared with contralateral healthy knee (*p* < 0.01), but only 33.2% (324/976) of the patients showed a moderate to severe degree (Grade C or D) of osteoarthritis.

#### Kellgren–Lawrence Grade

Analysing Kellgren–Lawrence Grade at final follow‐up, ACLR Group showed a higher degree of OA compared with contralateral healthy knee (*p* < 0.01), but only 28.9% (196/678) of the patients showed a moderate to severe degree (Grade III or IV) of osteoarthritis.

#### Ahlbäck Score

Analysing Ahlbäck score at final follow‐up, ACLR Group showed no difference regarding OA compared with contralateral healthy knee (*p* = 0.5), and 20.1% (33/164) of the patients showed a moderate to severe degree (Grade III or IV) of osteoarthritis. Detailed results are reported in Table 2.

**Table 2:** Radiographic outcomes, comparison with contralateral knee, failure rate, complications, and subsequent surgery analysed by graft.

Graft	Patients	Mean age (years)	Mean follow‐up (years)	% Failure rate (*n*)	Complications/subsequent surgery	Osteoarthritis	Contralateral knee	*p* Value (operated knee versus contralateral)
Patellar tendon	1290	24.97 ± 1.69	23.57 ± 2.59	9.45% (122)	Total: 18.3% (237) Infections 0.23% (3) Regional pain syndrome 0.31% (4) Stiffness 0.31% (4) Knee replacement surgery 1.16% (15) Meniscectomy 15.2% (197) Tibial osteotomies 0.62$ (8) Tibial screw removal 0.07% (1) Patellar tendon cyst 0.07% (1) Remove cyclops lesion 0.15% (2) Arthroscopy 0.15% (2)	IKDC grade (403): A 50% (163) B 21% (103) C 19% (86) D 10% (51) Kelllgren–Lawrence (249): Grade 0 19.2% (48) Grade 1 20.5% (51) Grade 2 29.3% (73) Grade 3 24.1% (60) Grade 4 6.8% (17) Ahlbäck (54) Grade 0 0% (0) Grade 1 57.4% (31) Grade II 18.5% (10) Grade III 22.2% (12) Grade IV 1.9%% (1)	IKDC grade (141): A 45.3% (64) B 41.8% (59) C 7.8% (11) D 4.9% (7) Kelllgren–Lawrence (224): Grade 0 57% (135) Grade 1 22% (52) Grade 2 8% (18) Grade 3 7% (17) Grade 4 1% (2) Ahlbäck (26) Grade 0 4% (1) Grade 1 77% (20) Grade II 15% (4) Grade III 4% (1) Grade IV 0% (0) Grade V 0% (0)	IKDC: *p* < 0.001* Kellgren–Lawrence: *p* < 0.001* Ahlbäck: *p* = 0.02*
Hamstring	190	26.2 ± 2.9	21.3 ± 1.68	13.15% (25)	Total: 24.2% (46) 11.05% (21) Mensicectomy 0.5% (1) Osteotomy 7.36% (14) Staples removal 1.6% (3) Arthroscopy 1% (2) Tibial screw removal 0.5% (1) Asportation cyclops lesion 2.1% (4) Paraesthesia	Kelllgren–Lawrence (90): Grade 0 11.1% (10) Grade 1 46.67% (42) Grade 2 18.89% (17) Grade 3 17.78% (16) Grade 4 5.56% (5) IKDC (61) A 59% (36) B 28% (17) C 7% (4) D 6% (4)	Kelllgren–Lawrence (39): Grade 0 49% (19) Grade 1 41% (16) Grade 2 10% (4) Grade 3 0% (0) Grade 4 0% (0)	Kellgren–Lawrence: *p* < 0.01*
Ilitiobial band	27	24.8	24	3.7% (1)	Total: 40.7% (11) Meniscectomy 37.0% (10) Arthroscopy due to stiffness 3.7% (1)	IKDC grade (24): A 8% (2) B 21% (5) C 25% (6) D 46% (11)	IKDC grade (24): A 42% (10) B 42% (10) C 8% (2) D 8% (2)	IKDC: *p* < 0.01*
Patellar tendon + LAD	45	27	25	15.6% (7)	Total: 13.3% (6) 13.3% (6) Arthroplasty	Ahlbäck (28) Grade 0 0% (0) Grade I 68% (19) Grade II 11% (3) Grade III 18% (5) Grade IV 3% (1) Grade V 0% (0)	NA	NA
Total	1552	25.18 ± 1.91	23.34 ± 2.56	9.98% (155)	19.3% (300)	IKDC grade (488): A 41.2% (201) B 25.6% (125) C 19.6% (96) D 13.5% (66) Kelllgren–Lawrence (339): Grade 0 17.1% (58) Grade 1 27.4% (93) Grade 2 26.5% (90) Grade 3 22.4% (76) Grade 4 6.4% (22) Ahlbäck (82) Grade 0 0% (0) Grade 1 60.9% (50) Grade II 15.8% (13) Grade III 20.7% (17) Grade IV 2.4%% (2)	IKDC grade (165): A 44.8%(74) B 41.8%(69) C 7.9%(13) D 5.5%(9) Kelllgren–Lawrence (263): Grade 0 58.5% (154) Grade 1 25.8% (68) Grade 2 8.4% (22) Grade 3 6.4% (17) Grade 4 0.7% (2) Ahlbäck (26) Grade 0 4% (1) Grade 1 77% (20) Grade II 15% (4) Grade III 4% (1) Grade IV 0% (0) Grade V 0% (0)	IKDC: *p* < 0.001* Kellgren–Lawrence: *p* < 0.001* Ahlbäck: *p* = 0.5

Abbreviations: IKDC, International Knee Documentation Committee; LAD, ligament augmentation device; NA, not available.

* Statistical significant value (*p* < 0.05).

## DISCUSSION

The main result of the current study demonstrated that ACL reconstruction appears to result in mild osteoarthritis in the long term in most of the patients and only 33.2% develop a moderate to severe degree of knee OA according to IKDC score. A slight degree of osteoarthritis appears to be present in ACLR knees compared with contralateral healthy knees.

Our results partially agree with current literature; recently, Grassi et al. review the outcomes, failure rate, incidence and predictors of OA for different ACLR techniques at a minimum 20‐year follow‐up. The authors concluded that most patients had satisfactory subjective outcomes 20 years after ACLR; however, abnormal anteroposterior or rotatory laxity was found in nearly 10% of cases. The presence of radiographic OA was high, especially in patients with concomitant meniscal or cartilage injuries, older age and delayed surgery; however, severe OA was present in only 12.8% of cases, and total knee arthroplasty was required in only 1.1% [20].

Everhart et al. in 2022 summarized outcomes at ≥20 years after ACL reconstruction and identified patient and surgical factors that affect these results. Five studies met the inclusion and exclusion criteria with a total of 2012 patients. Four studies (*n* = 584) reported graft tears at a mean rate of 11.8% (range: 2%–18.5%) and four studies (*n* = 773) reported a contralateral ACL injury rate of 12.2% (range: 5.8%–30%). Repeat non‐ACL arthroscopic surgery (four studies; *n* = 177) to the ipsilateral knee occurred in 10.4% (range, 9.5%–18.3%) and knee arthroplasty (one study; *n* = 217) in 5%. The IKDC objective score was normal or nearly normal in 82.3% (*n* = 496; three studies), with low rates of clinically significant residual laxity. Moderate‐severe radiographic osteoarthritis (OA) (IKDC grade C or D) was present in 25.9% of patients (*n* = 605; 3 studies) [16].

Similarly to our results, Claes et al. in 2013 reviewed the current literature on long‐term radiographic outcome after autologous ACL reconstruction. A total of 16 studies could be included for meta‐analysis, accounting for 1554 ACL reconstructions performed between 1978 and 1997. Of these knees, 453 (28%) showed radiological signs of osteoarthritis (IKDC grade C or D). The authors concluded that the prevalence of radiographic knee OA after ACL reconstruction is lower than commonly perceived [7].

Webster and Hewett studied the risk for the development and prevalence of knee OA after ACL injury and surgical treatment and compared prevalence rates between surgical and nonsurgical treatment. Combining all data from previous systematic reviews into a single source showed that ACL injury markedly increases the risk for the development of knee OA, which is likely to be present in the long term in approximately a third of patients who have reconstruction surgery. Surgical treatment does not reduce OA prevalence in the longer term compared with nonsurgical treatment [46].

When a graft analysis was performed, ITB showed the worst results in terms of osteoarthritis, with 71% of patients classified as IKDC C or D at radiographic follow‐up, which was significantly more frequent with respect to other grafts. However, the total number of patients who received this type of graft came from only one study, which referred to the prearthroscopic era. In fact, the patients in this study received combined intra‐articular and LET reconstruction with an open technique and postoperative immobilization in a cast. Additionally, in the IKDC evaluation of OA, patients who received PT grafts had a significantly higher percentage of moderate‐ to high‐grade OA than patients who received HT grafts (IKDC grades C and D—29% vs. 13%).

According to the current literature, there is no clear superiority of one ACL graft over another in terms of the long‐term incidence of OA.

In a multicentre study conducted by the Société Française de Chirurgie Orthopédique et de Traumatologie, the authors examined the long‐term impact of PT and HT graft techniques on the incidence of OA following ACLR. A total of 541 patients who underwent ACL reconstruction between 2002 and 2003 were included [22]. The primary outcome was the occurrence of moderate to severe osteoarthritis (IKDC C and D). The study found no significant difference in osteoarthritis rates between the PT and HS groups (19.3% for PT vs. 19.6% for HT, *p* = 0.94). Age over 29 years and an IKDC osteoarthritis stage B at initial surgery were identified as risk factors for OA progression. Interestingly, in patients requiring a medial meniscectomy, the HT group had a significantly higher rate of osteoarthritis, but this difference was not significant [22].

Barenius et al. conducted a randomized controlled trial aiming to evaluate the prevalence of OA after ACLR, comparing PT and HT grafts [2]. At the 14‐year follow‐up, 135 out of 164 patients underwent radiological assessments. The results showed that OA was significantly more common in the ACL‐reconstructed knee (57%) than in the contralateral knee (18%, *p* < 0.001). However, there was no significant difference in OA prevalence between the bone‐patellar tendon bone (49%) and semitendinosus (65%) grafts (*p* = 0.073). Additionally, meniscus resection was identified as a strong risk factor for OA, with an odds ratio of 3.6 (95% CI, 1.4–9.3). The study concluded that ACL reconstruction led to a threefold increase in OA prevalence compared with the contralateral knee, irrespective of graft type used. Meniscus resection was identified as a strong risk factor for OA, whereas the time between injury and reconstruction did not influence OA outcomes [2].

It is crucial to take into account various significant factors when analysing and understanding these findings. The surgical techniques and perspectives on ACLR discussed in this article are based on long‐term research conducted in the 1990s. It is important to note that these techniques may slightly differ from the current standard of practice. Concerning tunnel preparation, the positioning of the ACL femoral tunnel has changed since the time these procedures were conducted [11].

Rothrauff et al. reviewed the literature for radiographic prevalence of OA at a minimum of 10 years following ACLR with anatomic versus nonanatomic techniques. The authors concluded that anatomic ACLR was associated with lower OA prevalence at long‐term follow‐up [34].

A recent systematic review and meta‐analysis comprising 16 studies with a total of 1546 patients evaluated the influence of femoral tunnel positioning during ACLR on the development of OA [6]. The mean follow‐up time was 10.9 years. Two different techniques for ACLR were compared: the anteromedial and transtibial approaches. The study found that 49.3% of patients who underwent the transtibial technique developed radiographic OA, compared with 21.8% in the anteromedial group. Meta‐analysis showed a significantly higher rate of OA in the transtibial group across both 5‐ to 10‐year and greater than 10‐year follow‐up periods, concluding that ACLR using the transtibial approach was associated with higher overall rates of radiographic OA when compared with the anteromedial approach [6].

The current study found variations in rerupture rates and additional surgeries amongst different graft types. In terms of complications and subsequent surgery, the subanalysis of grafts showed significantly better values for PT and ITB grafts than for other grafts. Interestingly, patients who received ITB reconstruction had a significantly lower risk of graft failure than the other subgroups, although they had a higher incidence of complications and reinterventions.

In a large meta‐analysis conducted by Samuelsen et al. and including 47,613 patients, hamstring autografts had a slightly higher failure rate (2.84%) than bone‒tendon–bone autografts (2.80%), with an odds ratio of 0.83 (*p* = 0.01) [35]. However, both groups exhibited low failure rates, and there were few significant differences between the graft types in terms of laxity. The study concludes that both graft types are viable options for primary ACL reconstruction. The minor difference in failure rate is recommended to be part of a broader discussion with each patient, considering other factors like donor site morbidity, complication rates and patient‐reported outcome measures [35].

This study has several limitations. The inherent heterogeneity in the included studies, including differing surgical techniques and patient characteristics, made it impossible to conduct a meta‐analysis for different grafts. Additionally, the long‐term follow‐up duration could introduce recall bias or loss to follow‐up, potentially impacting the validity of the outcome measures. However, the methodological quality of the included studies, as measured by the MINORS score, was relatively high, providing confidence in the robustness of the findings. Variables that could have an impact on the incidence of OA, such as meniscectomy, concomitant procedures or the way in which the tunnel was performed, were not weighed. However, this possibility was precluded by the heterogeneity of the included studies, and therefore, we limited this study to a systematic review without performing a meta‐analysis. Finally, the small number of patients with PT grafts is another confusing factor and limitation for the study.

## CONCLUSIONS

ACL reconstruction appears to result in mild osteoarthritis in the long term in most of the patients and only less than 33.2% develop a moderate to severe degree of knee OA according to IKDC radiographic score. A slight degree of osteoarthritis appears to be present in ACLR knees compared with contralateral healthy knees.

## AUTHOR CONTRIBUTIONS

All authors contributed equally and approved the publication of this paper.

## CONFLICT OF INTEREST STATEMENT

The authors declare no conflict of interest.

## ETHICS STATEMENT

Ethical committee approval was not needed, being a literature review and not involving any human intervention. Consent to participate was not needed, being a systematic review and not involving any human intervention.

## Data Availability

The raw data are available with us, if needed. The datasets generated during and/or analysed during the current study are available from the corresponding author on reasonable request.
